# A rescue technique for segmental cholangitis after metallic stent placement in endoscopic ultrasound-guided hepaticogastrostomy

**DOI:** 10.1055/a-2883-0988

**Published:** 2026-06-23

**Authors:** Hidehito Sumiya, Shinsuke Koshita, Yoshihide Kanno, Takahisa Ogawa, Hiroaki Kusunose, Keisuke Yonamine, Kei Ito

**Affiliations:** 1 Gastroenterology 506803 Public Interest Incorporated Foundation Sendai City Medical CenterSendaiMiyagi PrefectureJapan

Endoscopic ultrasound-guided hepaticogastrostomy (EUS-HGS) using a fully covered self-expandable metallic stent (FCSEMS) can cause segmental cholangitis due to the obstruction of upstream bile ducts or intrahepatic branch ducts. 1​ ​2​ In addition, the removal of the FCSEMS before sufficient maturation of the endosonographically created route (ESCR) can carry a risk of bile leakage. We report a case of segmental cholangitis after EUS-HGS with FCSEMS placement that was managed by exchange to multiple plastic stents (PSs).

**Fig. 1:**
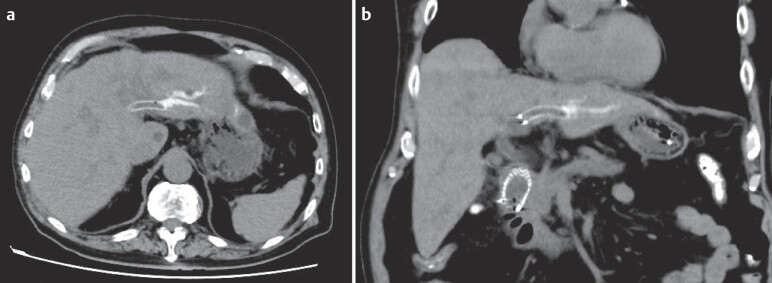
Computed tomographic images acquired the day after endoscopic ultrasound-guided hepaticogastrostomy. The upstream B2 bile duct and its peripheral branches were filled with contrast medium. ( **a** ) Horizontal image. ( **b** ) Coronal image.

An 86-year-old man with pancreatic head cancer underwent duodenal stent placement for duodenal stenosis, followed by EUS-HGS for obstructive jaundice. A FCSEMS (HANAROSTENT Biliary Full Cover Benefit, 8 mm×12 cm; Boston Scientific Japan K.K., Tokyo, Japan) was placed in the B2 bile duct across the ESCR. The day after EUS-HGS, the patient developed fever and abdominal pain. On computed tomography, the upstream B2 bile duct and its peripheral branches were filled with contrast medium, suggesting segmental cholangitis ( Fig. 1 ). Although conservative management was initiated, the cholangitis did not resolve. Therefore, stent exchange was performed 3 days after EUS-HGS.

**Fig. 2:**
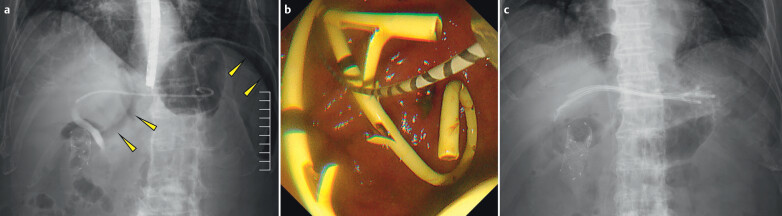
Endoscopic procedure for segmental cholangitis. ( **a** ) After the removal of the fully covered self-expandable metallic stent, pneumoperitoneum progressively worsened (arrowhead). ( **b** ) Three plastic stents were placed at the endosonographically created route. ( **c** ) The pneumoperitoneum gradually decreased.

After the removal of the FCSEMS, the gastric-side orifice of the ESCR was markedly dilated, and pneumoperitoneum was observed under fluoroscopy ( Video 1 and Fig. 2a ). After the placement of a 7-Fr PS (Through & Pass TYPE IT; Gadelius Medical K.K., Tokyo, Japan) across the ESCR, closure of the remaining gap at the orifice was attempted by using endoscopic clips. However, complete closure was technically difficult. To minimize the gap, two additional PSs (7- and 8.5-Fr) were placed across the ESCR ( Fig. 2b, c ). In addition, a nasogastric tube was inserted to minimize the leakage of the gastric juice through the ESCR. After the procedure, the cholangitis promptly resolved, and the patient was discharged without symptomatic bile leakage or other adverse events.

**Video 1** Demonstration of the rescue technique for segmental cholangitis after metallic stent placement in endoscopic ultrasound-guided hepaticogastrostomy.


Endoscopy_UCTN_Code_CPL_1AL_2AD
